# Image quality analysis of ^44^Sc on two preclinical PET scanners: a comparison to ^68^Ga

**DOI:** 10.1186/s40658-020-0286-3

**Published:** 2020-03-12

**Authors:** Florian Rosar, Hans-Georg Buchholz, Sebastian Michels, Manuela A. Hoffmann, Markus Piel, Christopher M. Waldmann, Frank Rösch, Stefan Reuss, Mathias Schreckenberger

**Affiliations:** 1 Department of Nuclear Medicine, University Medical Center of the Johannes Gutenberg-University Mainz, Mainz, Germany; 2 Department of Nuclear Medicine, Saarland University Medical Center, Homburg, Germany; 3 Institute of Nuclear Chemistry, Johannes Gutenberg-University Mainz, Mainz, Germany

**Keywords:** Scandium-44, Small animal PET, Phantom study, Resolution, Image quality, Positron energy, Positron range

## Abstract

**Background:**

^44^Sc has been increasingly investigated as a potential alternative to ^68^Ga in the development of tracers for positron emission tomography (PET). The lower mean positron energy of ^44^Sc (0.63 MeV) compared to ^68^Ga (0.83 MeV) can result in better spatial image resolutions. However, high-energy γ-rays (1157 keV) are emitted at high rates (99.9%) during ^44^Sc decay, which can reduce image quality. Therefore, we investigated the impact of these physical properties and performed an unbiased performance evaluation of ^44^Sc and ^68^Ga with different imaging phantoms (image quality phantom, Derenzo phantom, and three-rod phantom) on two preclinical PET scanners (Mediso nanoScan PET/MRI, Siemens microPET Focus 120).

**Results:**

Despite the presence of high-energy γ-rays in ^44^Sc decay, a higher image resolution of small structures was observed with ^44^Sc when compared to ^68^Ga. Structures as small as 1.3 mm using the Mediso system, and as small as 1.0 mm using the Siemens system, could be visualized and analyzed by calculating full width at half maximum. Full widths at half maxima were similar for both isotopes. For image quality comparison, we calculated recovery coefficients in 1–5 mm rods and spillover ratios in either air, water, or bone-equivalent material (Teflon). Recovery coefficients for ^44^Sc were significantly higher than those for ^68^Ga. Despite the lower positron energy, ^44^Sc-derived spillover ratio (SOR) values were similar or slightly higher to ^68^Ga-derived SOR values. This may be attributed to the higher background caused by the additional γ-rays. On the Siemens system, an overestimation of scatter correction in the central part of the phantom was observed causing a virtual disappearance of spillover inside the three-rod phantom.

**Conclusion:**

Based on these findings, ^44^Sc appears to be a suitable alternative to ^68^Ga. The superior image resolution makes it an especially strong competitor in preclinical settings. The additional γ-emissions have a small impact on the imaging resolution but cause higher background noises and can effect an overestimation of scatter correction, depending on the PET system and phantom.

## Background

^68^Ga is the most commonly used isotope in metal-organic radiotracers for positron emission tomography (PET). It is well established in the diagnostics of prostate cancer ([^68^Ga]Ga-PSMA-11) or neuroendocrine neoplasms ([^68^Ga]Ga-DOTA-TOC) [1, 2]. Due to its promising physical properties, ^44^Sc has been increasingly investigated as an alternative to ^68^Ga. The development of a ^44^Ti/^44^Sc generator system [3–5] and the possibility to produce ^44^Sc with a cyclotron using calcium targets [6–8] are poised to ensure the availability of the isotope.

^44^Sc decays by positron emission and electron capture at rates of 94.3% and 5.7%, respectively. The mean positron energy (Eβ_mean_) of ^44^Sc is 0.63 MeV and thereby lower than that of ^68^Ga (Eβ_mean_ = 0.83 MeV). As a result, the smaller positron range in tissue theoretically results in better spatial image resolutions. ^44^Sc is thus an interesting isotope that may be used in preclinical and clinical PET studies.

**Fig. 1:**
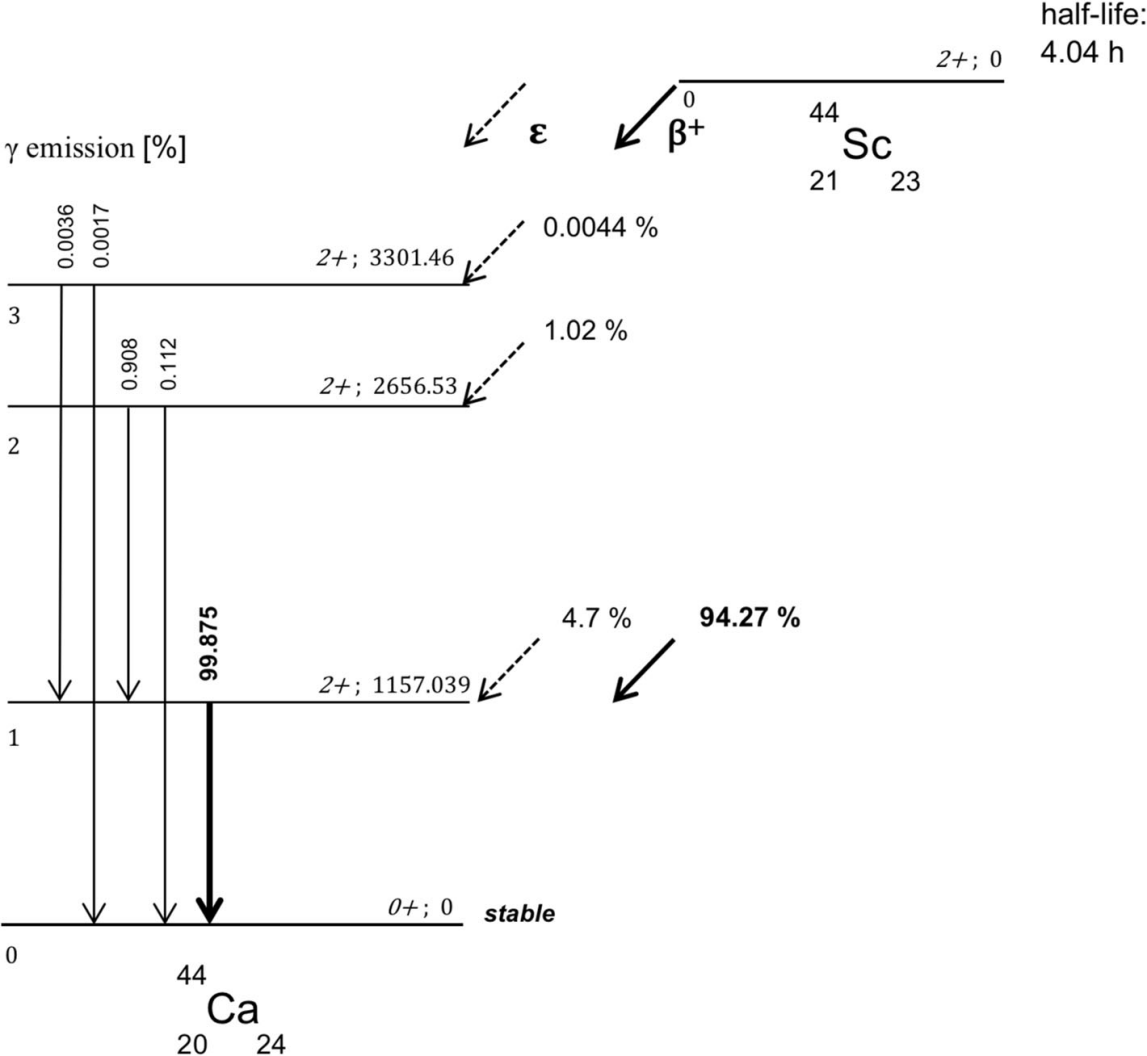
Primary and secondary transformation processes yielding ^44^Ca from ^44^Sc

Compared to ^68^Ga, ^44^Sc emits high-energy γ-rays (1157 keV) at high rates of 99.9% per decay during its stabilization process (Fig. 1). ^68^Ga emits γ-rays at low rates of 3.6%. Although these prompt γ-emissions occur above the energy window of a PET scan (typically 400–600 keV), they can appear in that window by losing energy through Compton scattering, thereby influencing imaging acquisition. As with other non-pure positron emitters such as ^86^Y or ^124^I, additional γ-emissions may lead to random coincidences as well as multiple coincidences of which both can cause reduced qualitative and quantitative imaging quality [9]. Most of these coincidences are insufficiently corrected by standard scatter- and attenuation correction methods. As a consequence, the background noise increases and a loss in contrast can be observed. Notably, these co-emissions can lead to higher radiation doses in patients and medical personnel.

The half-life of ^44^Sc is often described to be 3.97 h but a recent study reported a half-life of 4.04 h [10]. In contrast to ^68^Ga (half-life ~ 68 min), the ~ 4 h half-life of ^44^Sc allows to observe phenomena with slower kinetics such as the diffusion or excretion of larger biomolecules and metabolism processes. Furthermore, decentralized production of tracers and shipping to satellite PET centers are feasible with ^44^Sc but difficult to achieve with ^68^Ga [11, 12]. Table 1 compares the physical properties of ^44^Sc and ^68^Ga.

**Table 1:** Physical properties of ^44^Sc and ^68^Ga

	β^+^ rate	Mean β^+^ energy	Mean β^+^ range (*)	γ rate (**)	Mainly γ	Half-life
^44^Sc	94.3%	0.63 MeV	2.3 mm	> 99.9%	1157 keV	4.04 h
^68^Ga	89%	0.83 MeV	2.9 mm	3.6%	1077 keV	1.13 h

(*) in water/tissue, (**) in stabilization process

The coordination chemistry of Sc^3+^ is similar to that of other metal ions such as Lu^4+^ or Ga^3+^. Therefore, ^44^Sc binds to commonly used bifunctional chelating agents such as 1,4,7,10-Tetraazacyclododecane-1,4,7,10-tetraacetic acid (DOTA). Several in vitro and in vivo studies demonstrate the compatibility of ^44^Sc with different bifunctional DOTA-based chelating agents [13–17]. Experimental studies with [^44^Sc]Sc-DOTA-TATE or [^44^Sc]Sc-PSMA-617 showed similar characteristics (e.g., lipophilicity, binding affinity, and biodistribution) like the clinically used therapeutics [^177^Lu]Lu-DOTA-TATE and [^177^Lu]Lu-PSMA-617 [11, 17]. From a theragnostic point of view, the availability of the β^-^-emitter ^47^Sc (half-life = 3.35d) could allow the development of diagnostic/therapy tandems with chemically identical compounds [12, 18, 19]. In addition to preclinical studies on somatostatin analogues and PSMA ligands, ^44^Sc-labeled cetuximab F(ab’)_2_ fragments, HER2 affibodies, DOTA-NAPamides, and DOTA-Puromycins have been reported [14, 20–24]. Recently, the AAZTA scaffold was described to be a potent chelator for Sc^3+^ [25–27]. First clinical studies using [^44^Sc]Sc-DOTA-TOC and [^44^Sc]Sc-PSMA-617 underline the potential of ^44^Sc-labeled tracers as promising radiopharmaceuticals, especially when being used for pre-therapeutic dosimetry [28–30].

Only few preclinical phantom studies that investigate the resolution and image quality of ^44^Sc have been published so far [31, 32]. In an attempt to close this gap, we performed an unbiased complementary performance evaluation of ^44^Sc and ^68^Ga with three different imaging phantoms on two preclinical PET scanners. Typical image quality parameters, i.e., image resolution, recovery coefficient, and spillover ratios, were assessed for each scanner and isotope and the results were compared and discussed.

## Methods

To evaluate the performance with ^44^Sc on preclinical PET-systems, we performed multiple phantom studies on two preclinical PET scanners (Mediso nanoScan PET/MRI, Siemens Focus 120) with three different phantoms (image quality phantom, Derenzo phantom, three-rod phantom) and calculated several objective parameters for image resolution and image quality. The applied activity concentration of ^44^Sc ranged from 0.15 to 1.31 MBq/ccm depending on the phantom used. With decreasing rod diameter in the phantom, the activity concentration was increased. We performed analog experiments with ^68^Ga and the PET standard ^18^F. The particular activity concentrations of ^44^Sc and ^68^Ga are listed in Table 2 (values for ^18^F can be found in the supplementary material (Table S1)). For each phantom, the acquisition time was 30 min. In the following segment, the isotopes, the phantoms, and the PET scanners used in this study are briefly specified.

**Table 2:** Applied radioactivity of ^44^Sc and ^68^Ga

Phantom	PET system	Volume [ml]	^44^Sc activity concentration [MBq/ccm]	^68^Ga activity concentration [MBq/ccm]
Image quality	nanoScan PET/MRI	20	0.41	0.33
Image quality	microPET Focus 120	20	0.42	0.43
Derenzo	nanoScan PET/MRI	6	0.82	0.82
Derenzo	microPET Focus 120	6	1.31	1.20
Three-rod	nanoScan PET/MRI	93	0.15	0.24
Three-rod	microPET Focus 120	93	0.20	0.24

### Isotopes

#### ^44^Sc

^44^Sc was obtained from a ^44^Ti/^44^Sc generator that was developed at the Institute of Nuclear Chemistry at Mainz University, Germany [3]. Briefly, ~ 170 MBq of ^44^Sc were eluted with 20 mL of a mixture consisting of 0.07 M hydrochloric acid and 0.005 M oxalic acid. ^44^Sc was trapped on a cation exchange resin (AG 50 W-X8) and eluted with 3 mL of a 0.25 M ammonium acetate solution (pH 4). ~ 130 MBq of ^44^Sc were obtained containing less than 10 Bq of ^44^Ti-activity [4]. Quality control was performed using thin-layer chromatography (TLC) for chemical purity and gamma-spectroscopy for radionuclidic purity [5]. Measurement of ^44^Sc radioactivity was performed in a dose calibrator (VDC 405, Veenstra Instruments, Joure, Netherlands) with ^18^F-settings. A conversion factor of 0.70 to account for absolute ^44^Sc activity was applied [4].

#### ^68^Ga

^68^Ga was obtained from a ^68^Ge/^68^Ga generator based on a TiO_2_ matrix (Eckert & Ziegler AG, Berlin, Germany). Briefly, ~ 200 MBq of ^68^Ga were eluted with 5 mL of a 0.1 N hydrochloric acid solution. ^68^Ga was trapped on a cation exchange resin (AG 50 W-X8) and the resin rinsed with a solution consisting of 80% (v/v) acetone in 0.15 M hydrochloric acid solution [33]. The activity was eluted with a solution consisting of 97.56 (v/v) acetone in 0.05 M hydrochloric acid solution. ~ 180 MBq of ^68^Ga were obtained containing less than 0.00001% of ^68^Ge. Quality control was performed using TLC for chemical purity and gamma-spectroscopy for radionuclidic purity [34, 35].

### Phantoms

**Fig. 2:**
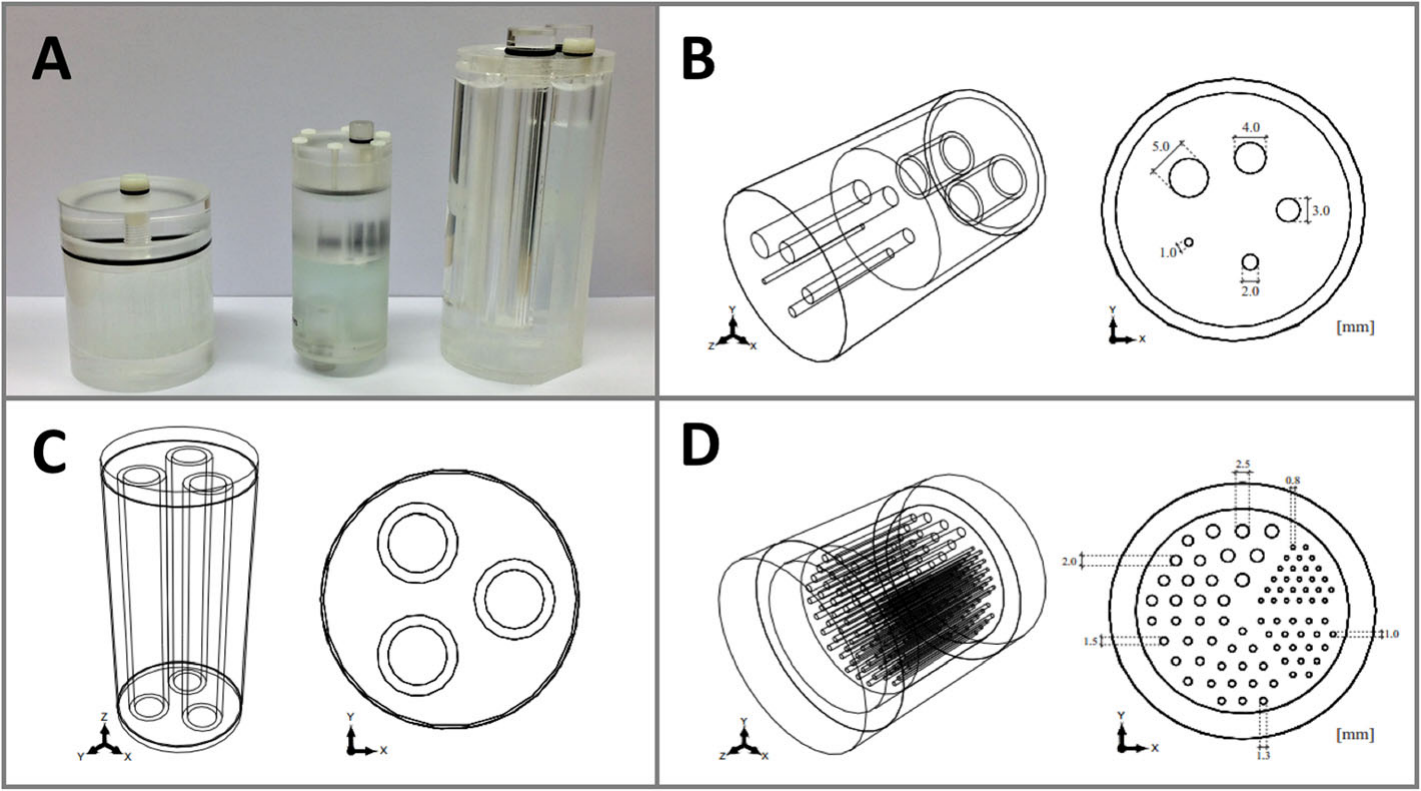
The phantoms being used (**a**). Schematic structure of image quality phantom (**b**), three-rod phantom (**c**), and Derenzo phantom (**d**)

The three phantoms used in this study were configured cylindrically with differently sized drilled holes and chambers (outlines in Fig. 2). All phantoms were made of polymethylmethacrylate to allow for their usage in PET/MRI scanners.

#### Image quality phantom

The image quality phantom is a standardized phantom (NEMA NU 4-2008) with a length of 63 mm and a diameter of 33.5 mm (Fig. 2b). It consists of a central main chamber that is connected to five rods in the front (*Ø* = 1, 2, 3, 4, 5 mm) which are located 7 mm around the center as well as two cylindrical chambers in the back (*Ø* = 8 mm). All three chambers can be filled separately and are not connected to each other. In this study, the main chamber was filled with the radioactive isotope and the two cylindrical chambers in the back were filled with air and water.

#### Three-rod phantom

The three-rod phantom is a custom-made phantom (based on NEMA NU 2-1994 standard) with a length of 107 mm and diameter of 48 mm (Fig. 2c). It consists of a main chamber and three cylindrical chambers (outside *Ø* = 15 mm, inside *Ø* = 11 mm) that are not connected to each other. The main chamber was filled with the radioactive isotope. Each cylindrical chamber was filled with either air, water, or Teflon. Teflon was used to simulate human bone tissue.

#### Derenzo phantom

The Derenzo phantom is a custom-made phantom similar to that described by Budinger et al. [36]. This cylindrical phantom comprises a length of 61 mm and an inside and outside diameter of 40 mm and 50 mm, respectively (Fig. 2d). A cylindrical block (*Ø* = 40 mm) with multiple small drills served as an insert. The differently sized rods (*Ø* = 0.8 mm, 1.0 mm, 1.3 mm, 1.5 mm, 2 mm, and 2.5 mm) are arranged in six sectors. The distance of two neighboring rods is twice the diameter (for diameters 0.8 –1.5 mm).

### PET scanners and reconstruction methods

#### Mediso nanoScan PET/MRI (Scanner 1)

The Mediso nanoScan PET/MRI, in the following referred to as “Scanner 1” is a hybrid small animal scanner consisting of a 12-detector block PET system and a 1-tesla MR system. Every detector block is composed of 39 × 81 lutetium-yttrium oxyorthosilicate (LYSO) crystals with dimensions of 1.12 mm × 1.12 mm × 13.00 mm (total crystal number 37908). The pitch totals 1.17 mm. The axial effective field of view spans 94 mm, whereas the transaxial span depends on the coincidence mode. Three different coincidence modes (1:1, 1:3, and 1:5) are available. Accordingly, the transaxial field of view spans 45, 94, or 120 mm. Each detector block is connected with two photomultipliers covered by a magnetic shield. The PET resolution is 700 μm according to the manufacturer. In this study, all data was obtained in coincidence mode 1:5 and subsequently reconstructed in coincidence mode 1:3. Analytical (2D-FBP, 3DRP) as well as iterative reconstructions (2D-OSEM, 3D-OSEM) are supported. In addition, Mediso offers an iterative reconstruction algorithm (TeraTomo-3D), which is based on a variation of 3D-OSEM/MAP using total variation as a penalty term weighed by a parameter α for regularization.

#### Siemens Focus 120 (Scanner 2)

The Siemens Focus 120, in the following referred to as “Scanner 2” is a non-hybrid small animal PET-scanner consisting of four ring detectors built by 96 detector blocks with 12 × 12 LSO crystals with 1.52 mm × 1.52 mm × 10 mm dimension (total crystal number 13824). The pitch totals 1.6 mm. The axial effective field of view spans 76 mm and the transaxial field of view spans 100 mm. Each block is connected with a 12-channel photomultiplier. The PET resolution stated by the manufacturer is ≤ 1400 μm with a volume resolution of 2.5 μl. For the Siemens Focus-120, analytical (2D-FBP, 3DRP) and iterative (2D-OSEM, 3D-OSEM, 3D-MAP, 3D-OSEM/MAP) reconstruction algorithms are available. The combined 3D-OSEM/MAP algorithm results in improved image resolution by taking scanner geometry and the point spread function of the detector response into account [37]. Additionally, a smoothing factor restricting values of neighboring voxels was applied.

In this study, an energy discrimination window of 400–600 keV on Scanner 1 and of 350–650 keV on Scanner 2 was set. For reconstruction, the TeraTomo-3D reconstruction algorithm (12 iterations, 6 subsets, 0.4 × 0.4 × 0.8 mm^3^ voxel size) with normal regularization (*α* = 0.0005) and 3D OSEM/MAP reconstruction algorithm (256 × 256 matrix, 2 OSEM iterations, 6 subsets, 18 MAP iterations, 0.4 × 0.4 × 0.4 mm^3^ voxel size) with a smoothing factor of 0.02 was applied. The used reconstruction algorithm with the abovementioned reconstruction settings were recommended by the vendors. All reconstructions included dead time correction, decay correction, normalization, and correction for random coincidence. Acquisition parameters as total coincidence rate, delayed random rate and dead time correction factors are compiled in the supplement material (Table S1). Scatter and attenuation correction were based on a MR-material-map obtained with the Mediso nanoScan PET/MRI or, at the Siemens Focus 120, on a prior transmission scan with ^57^Co.

### Parameters for image quality and image resolution

The following parameters were calculated based on volume-of-interest (VOI) data extracted by PMOD (version 3.606, PMOD Technologies LLC, Zürich, Switzerland).

### Recovery coefficient

The recovery coefficient (RC) is part of the National Electrical Manufacturers Association (NEMA) NU 4-2008 standard protocol and can be used to evaluate image quality. The RC describes the decrease of reconstructed activity compared to the true activity in smaller structures. This phenomenon is known as the partial volume effect (PVE), depends on the size of the PET crystals, and occurs if structures are smaller than 2.5 times of the detector size. According to the NEMA protocol, RC was assessed by the equation *Max*_*rod*_/*Mean*_*RV*_, while *Max*_*rod*_ was the maximum activity concentration in the averaged 10 mm central part of each radioactive rod of the image quality phantom, measured by circular region of interest. *Mean*_*RV*_ describes the measured mean activity concentration of a reference cylindrical VOI inside the main chamber.

### Spillover ratio

The spillover ratio (SOR) was calculated in the cold inserts of the three-rod phantom (air, water, and Teflon) and of the image quality phantom (air, water) by the equation *Mean*_*cold*_/*Mean*_*RV*._*Mean*_*cold*_ describes the measured mean activity concentration in a cylindrical volume in the non-radioactive (“cold”) area inside the rods with the half rod diameter according to NEMA standard protocols. *Mean*_*RV*_ describes an activity concentration of a reference VOI inside the main chamber. The error was calculated by the error propagation using standard deviations of average. In addition, SOR outside the phantom was measured in a hollow cylindrical volume around the three-rod phantom.

### Spatial resolution

The spatial resolution was measured by calculating the full width at half maximum (FWHM) in rods inside the Derenzo phantom. A line-profile across the center of representative inner rods was drawn on a transversal image. The profile was extracted and used to calculate the rod’s FWHM by curve fitting using a multi-parameter Gaussian function. Based on the FWHM calculation, both image quality (contrast) and image resolution (discrimination of two adjacent rods) were assessed. Goodness of fit was derived by using the standard errors of the fit parameters.

## Results

The results of the ^44^Sc and ^68^Ga measurements are presented in the following. All measurements were also performed with ^18^F and the results are presented in the supplementary material (Tables S2 – S4) as this study focusses on the analysis of ^44^Sc and the comparison with its chemical competitor ^68^Ga.

### Recovery coefficients

**Fig. 3:**
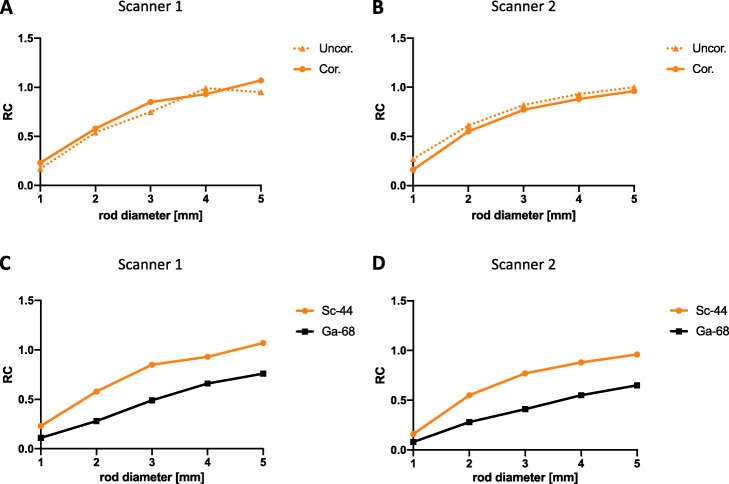
Recovery coefficient (RC) of ^44^Sc depending on rod diameter on both PET systems (**a**, **b**) with/without scatter and attenuation correction and comparison to ^68^Ga (**c**, **d**). The values are also compiled in the supplementary material (Table S2). Uncor. without scatter and attenuation correction, Cor. with scatter and attenuation correction

On both scanners, the measured RCs decreased with decreasing rod diameters. Without scatter and attenuation correction, the RC decreased from 0.95 in 5 mm rods to 0.17 in 1 mm rods on Scanner 1 and from 1.00 to 0.27 on Scanner 2, respectively. Linear interpolated RC curves of ^44^Sc on both scanners are shown in Fig. 3. With scatter and attenuation correction, a slight increase (mean: + 12.5%) of RC values was observed on Scanner 1 (Fig. 3a). The RC ranged from 1.07 in 5 mm rod to 0.23 in 1 mm rod. On Scanner 2, a slightly decrease (mean: − 13.2%) was observed when scatter and attenuation correction were applied (Fig. 3b). The RC ranged from 0.96 in 5 mm rod to 0.16 in 1 mm rod. In Fig. 3 c and d, calculated RC values of ^44^Sc are compared with RC values of ^68^Ga on both PET scanners. On both scanners, ^44^Sc revealed significantly higher RC-values than ^68^Ga with a mean difference of 40.8% on Scanner 1 and 43.1% on Scanner 2.

### Spillover ratios

#### SOR using three-rod phantom

**Fig. 4:**
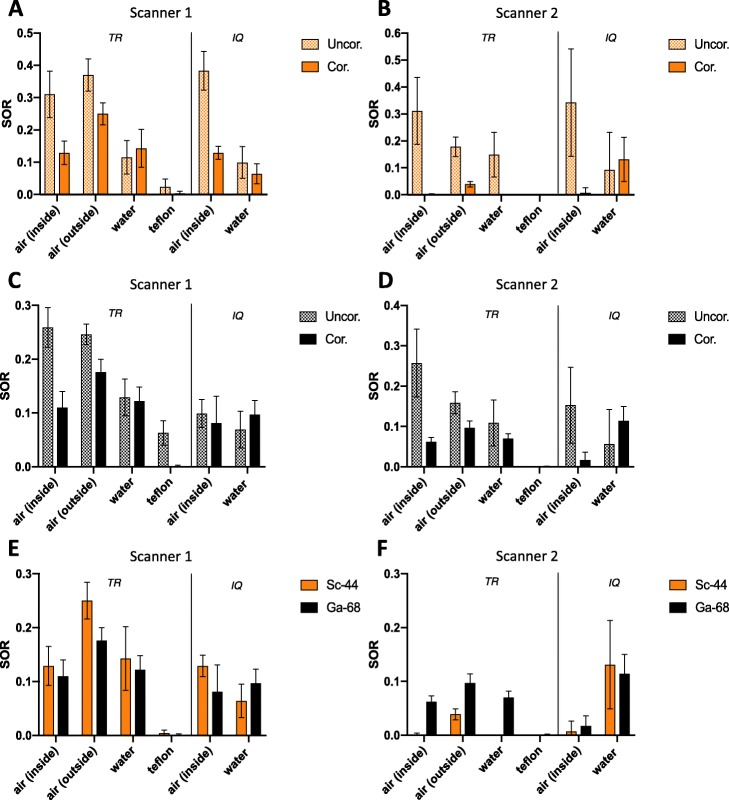
Spillover ratio (SOR) values of ^44^Sc (**a**, **b**) and ^68^Ga (**c**, **d**) with/without scatter and attenuation correction using three-rod (TR) and image quality (IQ) phantom on both PET systems and its comparison (**e**, **f**). Plotted are the calculated SOR values with their calculated errors. The values are also compiled in the supplementary material (Table S3). Uncor. without scatter and attenuation correction, Cor. with scatter and attenuation correction

**Fig. 5:**
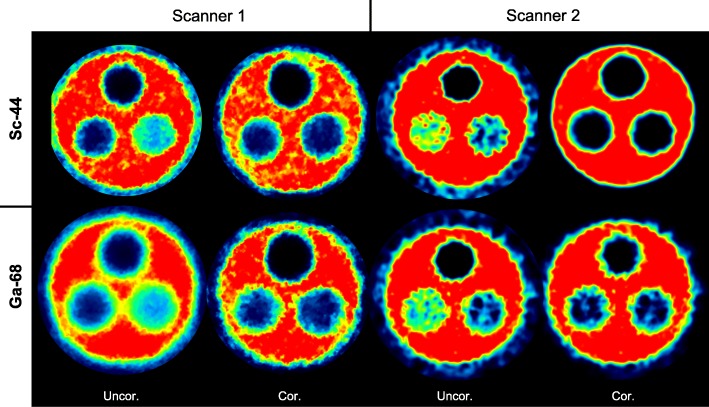
Transaxial slides of the three-rod phantom with ^44^Sc (first row) in comparison with ^68^Ga (second row) on both PET systems. A air, B water, C Teflon, Uncor. without scatter and attenuation correction, Cor. with scatter and attenuation correction

On both scanners, without scatter and attenuation correction, the highest SOR for ^44^Sc was observed in air followed by water and Teflon. The SOR in different media is illustrated in Fig. 4. When scatter and attenuation correction were applied on Scanner 1, SOR values decreased by 32.4% in air outside (0.370 to 0.250), by 58.4% in air inside (0.310 to 0.129) and by 83.3% in Teflon (0.024 to 0.004). No decrease was observed in water (Fig. 4a). In contrast, on Scanner 2, attenuation and scatter correction led to an extensive decrease in any medium (Fig. 4b). SOR decrease ranged from 78.1% in air outside the phantom (0.178 to 0.039), up to 100% (to 0.000) in any media inside the phantom. This extensive decrease of SOR was not observed using ^68^Ga. In case of the latter, the decrease ranges from 35.8% in water to 75.9% in air (Fig. 4d). A comparison of SOR values obtained with either ^44^Sc or ^68^Ga on both Scanner systems is shown in Fig. 4e and f. On Scanner 1, higher SORs were observed with ^44^Sc outside of the phantom. In all other non-radioactive inserts, no significant disparities in SORs were found (Fig. 4e) between ^44^Sc and ^68^Ga. In contrast, on Scanner 2, a significantly lower SOR was observed for ^44^Sc in any media (Fig. 4f). A visual confirmation of the observed SOR in different transaxial slices of the three-rod phantom is given in Fig. 5.

#### SOR using image quality phantom

On both scanners, using three-rod phantom without scatter and attenuation correction, higher SOR for ^44^Sc was observed in air than in water (Fig. 4). When scatter and attenuation correction were applied, SOR values decreased by 66.3% (0.383 to 0.129) in air and by 67.8% (0.199 to 0.064) in water on Scanner 1. On Scanner 2, attenuation and scatter correction led to a substantial decrease of SOR in air by 98.0% (0.342 to 0.07) and to no decrease of SOR in water. As shown in Fig. 4 e and f, no relevant disparities in SOR between ^44^Sc and ^68^Ga were observed on either scanner.

### Spatial resolution

**Fig. 6:**
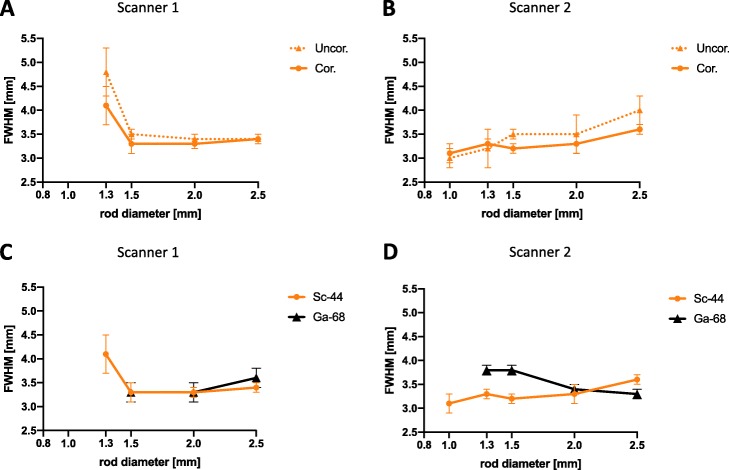
Full width at half maximum (FWHM) of ^44^Sc depending on rod diameter on both PET systems (**a**, **b**) with/without scatter and attenuation correction and comparison to ^68^Ga (**c**, **d**). Plotted are the calculated FWHM values with their calculated errors. The values are also compiled in the supplementary material (Table S4). Uncor. without scatter and attenuation correction, Cor. with scatter and attenuation correction

**Fig. 7:**
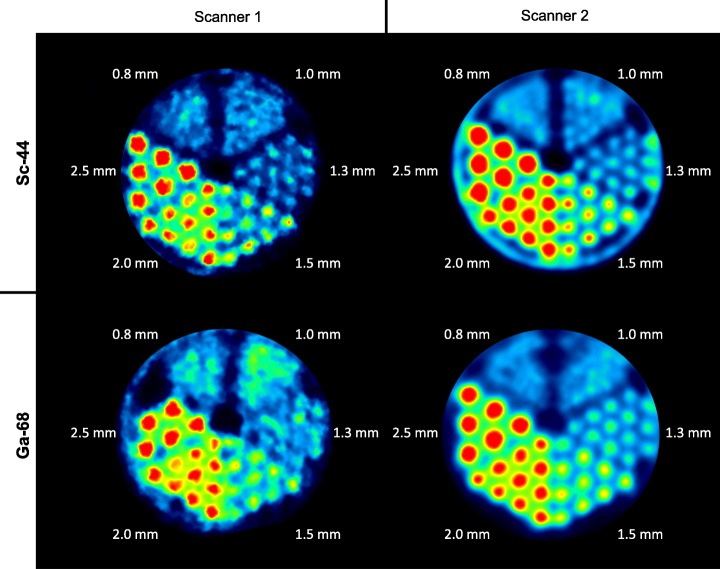
Summed up transaxial slices of the Derenzo phantom with ^44^Sc in comparison to ^68^Ga on both scanners

In case of ^44^Sc, structures as small as 1.3 mm or 1.0 mm can be visualized using Scanner 1 or Scanner 2, respectively. The FWHM dependency on rod diameter on both scanners is shown in Fig. 6. By applying scatter and attenuation correction, the FWHM could be partly decreased or remained similar. On Scanner 2, a steady decrease of the FWHM from 2.5 to 1.0 mm rod diameter was observed. In contrast, on Scanner 1, the FWHM was similar in the range from 2.5 to 1.5 mm rod diameter but a remarkable increase was overserved in the range from 1.5 to 1.0 mm. In comparison to ^68^Ga, smaller structures were identified using ^44^Sc. On Scanner 1, structures of sizes up to 1.3 mm were identified with ^44^Sc and structures of sizes up to 1.5 mm were identified with ^68^Ga. On Scanner 2, structures of sizes up to 1.0 mm were identified with ^44^Sc and structures of sizes up to 1.3 mm were identified with ^68^Ga. The FWHM calculations for Scanner 1 resulted in similar values for both nuclides. Significantly, lower values for ^44^Sc were calculated for Scanner 2 in case of rod diameters ≤ 1.5 (Fig. 6d). Summed transversal slices of the Derenzo phantom with ^44^Sc and ^68^Ga are depicted in Fig. 7.

## Discussion

### Recovery coefficients

**Fig. 8:**
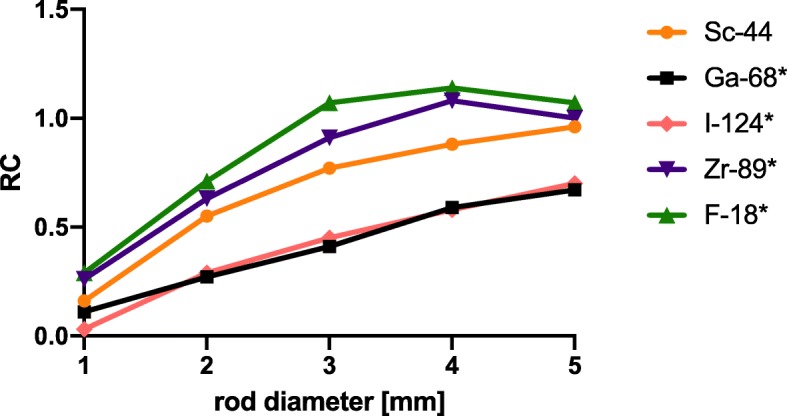
Comparison of our RC-values of ^44^Sc (Eβ_mean_ = 0.63 MeV) to RC-values of ^18^F (Eβ_mean_ = 0.25 MeV), ^89^Zr (Eβ_mean_ = 0.40 MeV), ^124^I (Eβ_mean_ = 0.82 MeV), and ^68^Ga (Eβ_mean_ = 0.83 MeV) by Disselhorst et al. 2010 (*). (reconstruction algorithm: 3D-OSEM/MAP)

The decrease of measured RCs of ^44^Sc with decreasing rod diameters may be explained by the impact of the positron range as well as the partial volume effect. Due to the positron range, a spatial distribution of the reconstructed activity that is larger than that of the geometrical rod diameter is observed. Thus, compared to the true activity, the reconstructed activity decreases with smaller rod diameter, leading to smaller RC values. On both scanners, RC values for ^44^Sc (Eβ_mean_ = 0.63 MeV) were significantly higher than those for ^68^Ga (Eβ_mean_ = 0.83 MeV). This expected behavior can be explained by the different positron energies and the resulting different positron ranges. A higher positron range leads to a larger distribution of the reconstructed activity and thus a lower RC. To the best of our knowledge, a comparable assessment of RCs for ^44^Sc has not been published to date. In Fig. 8, the RC values for ^44^Sc, as obtained from Scanner 2 in this study, are compared to RC values of different isotopes (^18^F, ^68^Ga, ^89^Zr, ^124^I) that were reported by Disselhorst et al. using similar reconstruction methods [38]. The RC values obtained for ^68^Ga in this study are similar to those reported by Disselhorst et al., which indicates that a comparison of both datasets is feasible*.* As expected, the RC values of ^44^Sc (Eβ_mean_ = 0.63 MeV) range between ^89^Zr (Eβ_mean_ = 0.40 MeV) and ^124^I (Eβ_mean_ = 0.82 MeV). It can be concluded that RC values mostly depend on the positron energy. Furthermore, additional γ-emissions by ^44^Sc appear to play a minor role in the determination of RC values but cause larger error intervals.

### Spillover ratios

**Fig. 9:**
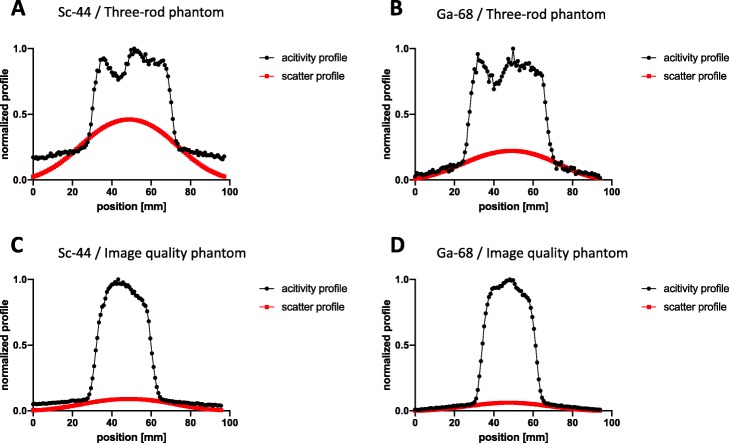
Normalized activity and scatter profile for both nuclides using three-rod phantom (**a**: ^44^Sc, **b**: ^68^Ga) and image quality phantom (**c**: ^44^Sc, **d**: ^68^Ga) on Scanner 2 (normalization to the maximum of the shown projection)

Without scatter and attenuation correction, the highest SOR was expectedly observed in air, followed by water and Teflon. This observation can be explained by the differences in material densities that influence positron deceleration. Applying scatter and attenuation correction on Scanner 1 caused a decrease in SOR values in both phantoms. ^44^Sc-derived SOR values obtained from air inside, water, and Teflon were similar or slightly higher (in case of air outside) to ^68^Ga-derived SOR values. This is surprising since ^44^Sc has a smaller positron energy. This finding likely results from the additional γ-emission during ^44^Sc decay. The γ-emission might also explain the higher error intervals observed for ^44^Sc. The γ-emission increased the background noise, which also led to an increase in measured activity in areas that are apparently free of activity. This phenomenon was described in previous studies for other non-pure positron emitters such as ^124^I, ^76^Br, and ^86^Y [39–41]. To the best of our knowledge, no SOR values for ^44^Sc have been reported to date in literature. On Scanner 2, we observed a significant decrease of SOR values with a virtual disappearance of spillover inside the three-rod phantom. This was caused by overestimation of scatter correction that results from additional γ-emissions. In Fig. 9 a and b, the normalized activity and scatter profiles of ^44^Sc and ^68^Ga in the three-rod phantom are compared. They differ in the activity profile with significantly higher radial tails for ^44^Sc due to higher background noise caused by γ-emissions. The reconstruction software of Scanner 2 offers a possibility to adjust the scatter correction for none-pure PET isotopes by modification of the tail fitting for scatter estimation. The standard correction factor in this modification was tested for ^86^Y but did not fit for ^44^Sc in this case. As shown in Fig. 9 a, when using this standard setting, the simulated scatter profile modeled to the ^44^Sc raw data was too high, especially in the center part of the three-rod phantom. This overestimation of calculated scatter for non-pure positron emitters led to over-subtraction in the central part. Using the image quality phantom, the overcorrection was not as prominent as with the three-rod phantom (Fig. 9c, d), which may be attributed to geometrical differences of the phantoms. This issue is also noted in the literature and consequently, different approaches for prompt γ-corrections have been proposed [9]. A suitable correction of additional γ-emissions is necessary and should be applied in further phantom studies.

### Spatial resolution

In general, observed image resolutions obtained with ^44^Sc were superior to those obtained with ^68^Ga. On both PET systems, smaller rod diameters could be visualized and analyzed with ^44^Sc. ^44^Sc also showed significantly smaller FWHM than ^68^Ga in rods with small diameters. This indicates that the spatial resolution is dependent more on the positron energy and the resulting positron range than on prompt γ-emissions. This finding is consistent with the results of several previous phantom studies [31, 38, 42, 43]. In Table 3, the maximum spatial image resolutions in a Derenzo phantom of ^44^Sc, ^68^Ga, and of other nuclides are compiled [42, 43]. These previously published data fit well with our data and support the assumption that positron energy and scanner properties are the main factors for the maximum spatial resolution of preclinical PET systems. Despite the better NEMA intrinsic resolution of Scanner 1 that is caused by smaller crystals, tomographic resolution of Scanner 2 was slightly superior. This may be explained by including the resolution recovery in the OSEM3D/MAP reconstruction of Scanner 2 and applying dedicated point spread functions.

**Table 3:** Compilation of the maximum resolution of different nuclides from different phantom studies using the Derenzo phantom

Isotope	Eβ_mean_	γ	Max. resolution	PET system
^52^Mn	0.24 MeV	> 100%	1.0 mm^*^	Inveon microPET
^18^F	0.25 MeV	~ 0%	0.8 mm	mircoPET Focus120-nanoScan PET/MRI
^64^Cu	0.28 MeV	0.47%	1.0 mm^*^	Inveon microPET
^89^Zr	0.40 MeV	~ 100%	1.0 mm^#^–1.25 mm^*^	microPET Focus 120 Inveon microPET
^44^Sc	0.63 MeV	~ 100%	1.0–1.3 mm	microPET Focus 120-nanoScan PET/MRI
^124^I	0.82 MeV	87.4%	1.5 mm^*^	Inveon microPET
^68^Ga	0.83 MeV	3.6%	1.3–1.5 mm	microPET Focus-120-nanoScan PET/MRI

(*Cox et al. 2016, [43] ^#^Laforest et al. 2008 [42])

Compared to the only phantom study in literature by Bunka et al. that investigated the spatial resolution of ^44^Sc [31], we calculated higher FWHM values (discrepancy 43.4-78.2%) which can be explained by different experimental settings (different phantoms, different PET scanners, different reconstruction algorithms) and different analyses (different line profiles, different fit curves, background subtraction). Nevertheless, Bunka et al. also reported smaller FWHM for ^44^Sc than for ^68^Ga, which is in accordance with our finding that ^44^Sc enables imaging with improved resolution.

Based on this image analysis, ^44^Sc appears to be a suitable alternative to ^68^Ga. Representative in vivo images are shown in the supplementary section (Figure S1). The animal studies will be reported elsewhere. This phantom study is limited by using only iterative 3D reconstruction algorithms and preselected reconstruction parameters. The argument that ^44^Sc is a suitable alternative to ^68^Ga with superior image resolution and recovery, may be true with different reconstruction algorithms as Bunka et al. observed using OSEM-2D [31]. Further studies investigating the optimal reconstruction and reconstruction parameters for ^44^Sc are needed as standard reconstruction settings used in this study were optimized for ^18^F. In comparison to ^18^F, ^44^Sc showed an expected inferior image quality caused by the ~ 2.5 times higher positron energy (0.63 MeV vs. 0.25 MeV).

The additional γ-emissions not only cause higher background noise and affect scatter correction; they also result in higher radiation dose for the animal or patient. Eppard et al. reported a two times higher absorbed kidney dose (mSv/MBq) with ^44^Sc than with ^68^Ga labeled PSMA-617 in patients with metastasized castration-resistant prostate carcinoma [29]. However, by taking advantage of the longer half-life, applied activity could be reduced in many cases. Eppard et al. administrated 50 MBq of ^44^Sc-PSMA-617 and achieved a comparable image quality as with 120 MBq of ^68^Ga-PSMA-11 [29]. The higher radiation dose for the technical staff also has to be taken into account. Further radiation shield to protect technical staff may be needed.

## Conclusions

Based on this image analysis, ^44^Sc appears to be a suitable alternative to ^68^Ga. The superior image resolutions obtained with ^44^Sc that is caused by its lower positron energy makes it a strong competitor, especially in a preclinical setting. Additional γ-emissions are causing higher background noises and an overestimation of scatter correction in the central part of the phantom, depending on the PET system and the phantom. These additional γ-emissions should be corrected for in further studies to insure they are not distorting image corrections and to allow for quantification of the PET-signal.

## Supplementary information

**Additional file 1.** Supplement tables and figures.

## Data Availability

The datasets used and analyzed during the current study are available from the corresponding author on reasonable request.

## Abbreviations

3DRP: 3D reprojection reconstruction
Cor.: With scatter and attenuation correction
DOTA: 1,4,7,10-Tetraazacyclododecane-1,4,7,10-tetraacetic acid
Eβ_mean_: Mean positron energy
FBP: Filtered back projection
FWHM: Full width at half maximum
IQ: Image quality phantom
LYSO: Lutetium-yttrium oxyorthosilicate
MAP: Maximum a posteriori image reconstruction
MRI: Magnetic resonance imaging
NEMA: National Electrical Manufactures Association
OSEM: Ordered subset expectation maximization
PET: Positron emission tomography
PSF: Point spread function
PSMA: Prostate specific membrane antigen
PVE: Partial volume effect
RC: Recovery coefficient
Scanner 1: Mediso nanoScan PET/MRI
Scanner 2: Siemens Focus 120
SOR: Spillover ratio
TLC: Thin-layer chromatography
TR: Three-rod phantom
Uncor: Without scatter and attenuation correction
VOI: Volume of interest
