# Supplementary material for: Image quality analysis of 44Sc on two preclinical PET scanners: a comparison to 68Ga
Source: EJNMMI Phys. 2020 Mar 12;7:16. doi: 10.1186/s40658-020-0286-3 (PMC7067939; doi:10.1186/s40658-020-0286-3)
Supplement: Supplementary file 1 — Additional file 1. Supplement tables and figures. [file 40658_2020_286_MOESM1_ESM.docx]

**Additional file 1: Supplement tables and figures**

**a) Acquisition parameters**

**Table S1:** Information of applied activities and acquisition parameters.

| PET system | Phantom | *Volume [ml]* | Phantom | *Activity [MBq]* | *Activity concentration [MBq/ml]* | *Total coincidence rate [kcps]* | *Delayed random rate [kcps]* | *Dead time correction* | |
| --- | --- | --- | --- | --- | --- | --- | --- | --- | --- |
|  |  |  |  |  |  |  |  | *Correction factor* | *Pile-Up factor* |
| nanoScan  PET/MRI | Image quality | 20 | ^44^Sc | 8.2 | 0.41 | 700 | 35 | - | 0.62 |
|  |  |  | ^68^Ga | 6.6 | 0.33 | 500 | 21 | - | 0.78 |
|  |  |  | ^18^F | 6.0 | 0.30 | 492 | 19 | - | 0.80 |
|  | Three-rod | 93 | ^44^Sc | 13.5 | 0.15 | 830 | 78 | - | 0.76 |
|  |  |  | ^68^Ga | 22.0 | 0.24 | 900 | 160 | - | 0.66 |
|  |  |  | ^18^F | 23.8 | 0.26 | 960 | 185 | - | 0.63 |
|  | Derenzo | 6 | ^44^Sc | 4.9 | 0.82 | 375 | 11 | - | 0.85 |
|  |  |  | ^68^Ga | 4.9 | 0.82 | 370 | 12 | - | 0.84 |
|  |  |  | ^18^F | 11.4 | 1.90 | 750 | 52 | - | 0.60 |
| microPET  Focus 120 | Image quality | 20 | ^44^Sc | 8.3 | 0.42 | 200 | 9.1 | 1.037 | - |
|  |  |  | ^68^Ga | 8.6 | 0.43 | 202 | 11.6 | 1.032 | - |
|  |  |  | ^18^F | 8.2 | 0.41 | 193 | 11.6 | 1.028 | - |
|  | Three-rod | 93 | ^44^Sc | 13.3 | 0.20 | 218 | 18 | 1.051 | - |
|  |  |  | ^68^Ga | 22.0 | 0.24 | 296 | 5.6 | 1.180 | - |
|  |  |  | ^18^F | 16.8 | 0.18 | 250 | 37 | 1.060 | - |
|  | Derenzo | 6 | ^44^Sc | 5.5 | 1.31 | 125 | 3.5 | 1.022 | - |
|  |  |  | ^68^Ga | 7.2 | 1.20 | 128 | 4.7 | 1.013 | - |
|  |  |  | ^18^F | 5.9 | 0.99 | 130 | 4.5 | 1.019 | - |

**b) Image parameters**

i) Recovery coefficient (RC)

**Table S2:** Calculated RC values (image quality phantom).

| PET system | Nuclide | Reconstruction algorithm | *Scatter and attenuation correction* | *Rod diameter* | | | | |
| --- | --- | --- | --- | --- | --- | --- | --- | --- |
|  |  |  |  | *5 mm* | *4 mm* | *3 mm* | *2 mm* | *1 mm* |
| nanoScan  PET/MRI | ^44^Sc | TeraTomo-3D | + | 1.07 | 0.93 | 0.85 | 0.58 | 0.23 |
|  |  | TeraTomo-3D | - | 0.95 | 0.99 | 0.75 | 0.54 | 0.17 |
|  | ^68^Ga | TeraTomo-3D | + | 0.76 | 0.66 | 0.49 | 0.28 | 0.11 |
|  |  | TeraTomo-3D | - | 0.89 | 0.78 | 0.60 | 0.33 | 0.12 |
|  | ^18^F | TeraTomo-3D | + | 1.25 | 1.27 | 1.17 | 0.94 | 0.31 |
|  |  | TeraTomo-3D | - | 1.49 | 1.43 | 1.42 | 1.11 | 0.33 |
| microPET  Focus 120 | ^44^Sc | 3D OSEM/MAP | + | 0.96 | 0.88 | 0.77 | 0.55 | 0.16 |
|  |  | 3D OSEM/MAP | - | 1.00 | 0.93 | 0.82 | 0.61 | 0.27 |
|  | ^68^Ga | 3D OSEM/MAP | + | 0.65 | 0.55 | 0.41 | 0.28 | 0.08 |
|  |  | 3D OSEM/MAP | - | 0.50 | 0.43 | 0.33 | 0.21 | 0.08 |
|  | ^18^F | 3D OSEM/MAP | + | 1.12 | 1.02 | 0.95 | 0.87 | 0.30 |
|  |  | 3D OSEM/MAP | - | 1.09 | 1.06 | 0.97 | 0.80 | 0.33 |

ii) Spillover ratio (SOR)

**Table S3:** Calculated SOR values (three-rod phantom and image quality phantom).

| PET system | Nuclide | Reconstruction algorithm | *Scatter and attenuation correction* | *Three-rod phantom* | | | | *Image quality phantom* | |
| --- | --- | --- | --- | --- | --- | --- | --- | --- | --- |
|  |  |  |  | *Air (inside)* | *Air (outside)* | *Water* | *Teflon* | *Air (inside)* | *Water* |
| nanoScan  PET/MRI | ^44^Sc | TeraTomo-3D | + | 0.129±0.036 | 0.250±0.034 | 0.143±0.006 | 0.004±0.006 | 0.129±0.020 | 0.064±0.031 |
|  |  | TeraTomo-3D | - | 0.310±0.072 | 0.370±0.050 | 0.115±0.052 | 0.024±0.026 | 0.383±0.060 | 0.199±0.049 |
|  | ^68^Ga | TeraTomo-3D | + | 0.110±0.030 | 0.176±0.024 | 0.122±0.026 | 0.001±0.002 | 0.081±0.050 | 0.097±0.026 |
|  |  | TeraTomo-3D | - | 0.259±0.037 | 0.246±0.019 | 0.129±0.034 | 0.063±0.023 | 0.099±0.026 | 0.069±0.034 |
|  | ^18^F | TeraTomo-3D | + | 0.075±0.029 | 0.075±0.009 | 0.075±0.029 | 0.000±0.000 | 0.029±0.010 | 0.031±0.010 |
|  |  | TeraTomo-3D | - | 0.217±0.045 | 0.104±0.012 | 0.047±0.014 | 0.006±0.005 | 0.132±0.026 | 0.026±0.007 |
| microPET  Focus 120 | ^44^Sc | 3D OSEM/MAP | + | 0.000±0.004 | 0.039±0.010 | 0.000±0.000 | 0.000±0.000 | 0.007±0.019 | 0.131±0.082 |
|  |  | 3D OSEM/MAP | - | 0.331±0.124 | 0.178±0.036 | 0.149±0.083 | 0.000±0.000 | 0.342±0.199 | 0.092±0.140 |
|  | ^68^Ga | 3D OSEM/MAP | + | 0.062±0.011 | 0.097±0.017 | 0.070±0.012 | 0.002±0.002 | 0.017±0.019 | 0.114±0.036 |
|  |  | 3D OSEM/MAP | - | 0.257±0.084 | 0.159±0.027 | 0.109±0.057 | 0.000±0.000 | 0.153±0.094 | 0.057±0.085 |
|  | ^18^F | 3D OSEM/MAP | + | 0.049±0.031 | 0.017± 0.002 | 0.027±0.021 | 0.005±0.008 | 0.014±0.018 | 0.010±0.011 |
|  |  | 3D OSEM/MAP | - | 0.241±0.076 | 0.066± 0.008 | 0.080±0.032 | 0.000±0.000 | 0.205±0.060 | 0.000±0.001 |

iii) Full width at half maximum (FWHM)

**Table S4:** Calculated FWHM values (Derenzo phantom).

| PET system | Nuclide | Reconstruction algorithm | *Scatter and attenuation correction* | *Rod diameter* | | | | | |
| --- | --- | --- | --- | --- | --- | --- | --- | --- | --- |
|  |  |  |  | *2.5 mm* | *2.0 mm* | *1.5 mm* | *1.3 mm* | *1.0 mm* | *0.8 mm* |
| nanoScan PET/MRI | ^44^Sc | TeraTomo-3D | + | 3.4±0.1 | 3.3±0.1 | 3.3±0.2 | 4.1±0.4 | - | - |
|  |  | TeraTomo-3D | - | 3.4±0.1 | 3.4±0.1 | 3.5±0.1 | 4.8±0.5 | - | - |
|  | ^68^Ga | TeraTomo-3D | + | 3.6±0.2 | 3.3±0.2 | 3.3±0.2 | - | - | - |
|  |  | TeraTomo-3D | - | 3.1±0.3 | 3.2±0.3 | 3.0±0.2 | - | - | - |
|  | ^18^F | TeraTomo-3D | + | 2.3±0.2 | 1.8±0.1 | 1.6±0.1 | 1.6±0.1 | 1.7±0.1 | 2.1±0.1 |
|  |  | TeraTomo-3D | - | 2.1±0.1 | 1.9±0.1 | 1.9±0.1 | 1.8±0.1 | 1.6±0.1 | 2.2±0.1 |
| microPET  Focus 120 | ^44^Sc | 3D OSEM/MAP | + | 3.6±0.1 | 3.3±0.2 | 3.2±0.1 | 3.3±0.1 | 3.1±0.2 | - |
|  |  | 3D OSEM/MAP | - | 4.0±0.3 | 3.5±0.4 | 3.5±0.1 | 3.2±0.4 | 3.0±0.2 | - |
|  | ^68^Ga | 3D OSEM/MAP | + | 3.3±0.1 | 3.4±0.1 | 3.8±0.1 | 3.8±0.1 | - | - |
|  |  | 3D OSEM/MAP | - | 3.4± 0.1 | 3.2±0.2 | 3.3±0.1 | 3.1±0.2 | - | - |
|  | ^18^F | 3D OSEM/MAP | + | 2.2± 0.0 | 1.8±0.0 | 1.6±0.0 | 1.6±0.0 | 1.9±0.0 | 1.9±0.1 |
|  |  | 3D OSEM/MAP | - | 2.3± 0.1 | 1.9±0.0 | 1.6±0.0 | 1.5±0.0 | 1.8±0.0 | 1.9±0.1 |

All values in mm.

**c) In-vivo images**

**
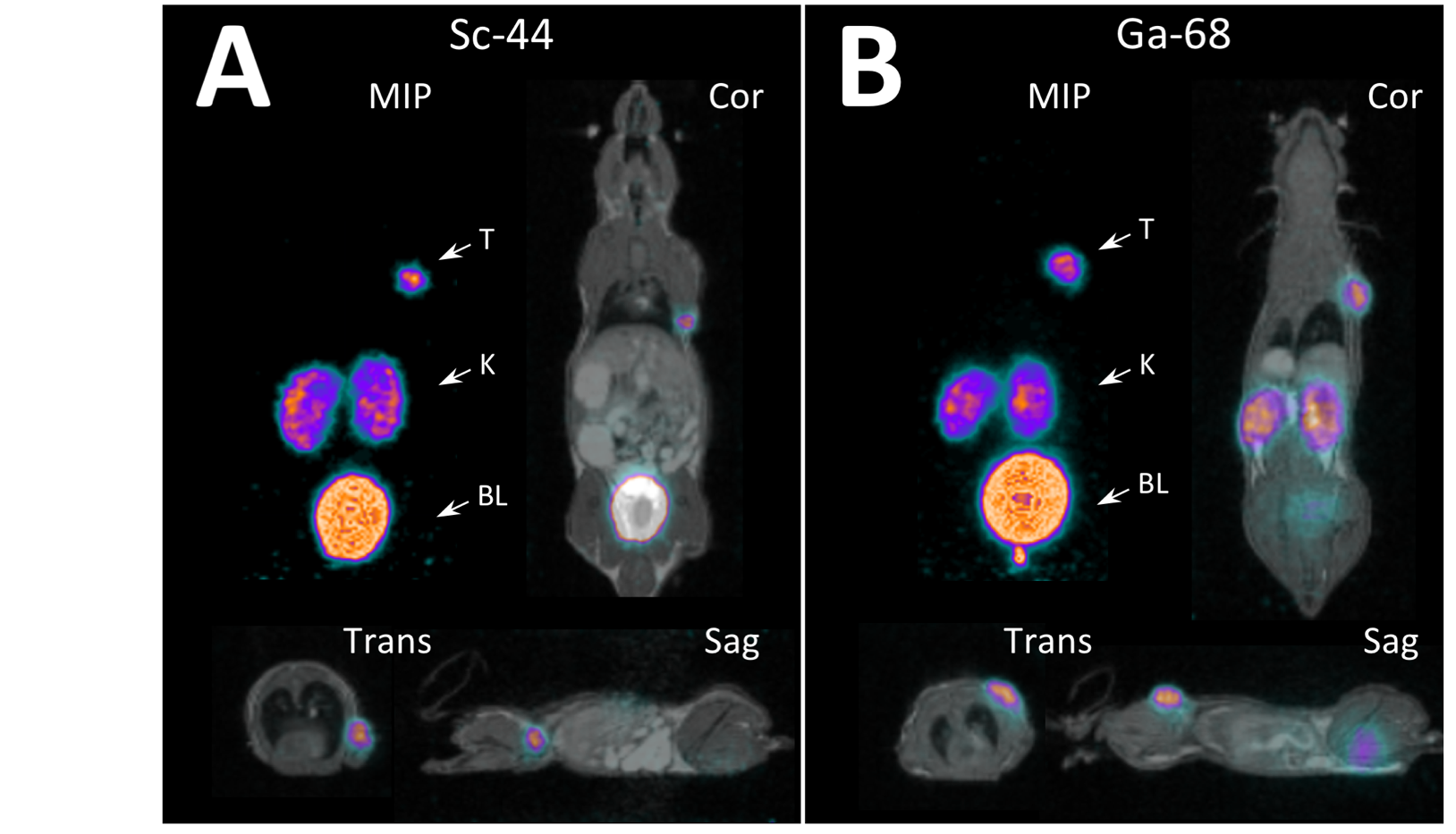
**

**Figure S1**: PET/MRI images (maximum intensity projection (MIP), coronal slice (Cor), transaxial slice (Trans) and sagittal slice (Sag)) with ^44^Sc (A) and ^68^Ga (B) labeled PSMA-ligands in two individual BALB/c-nu/nu mice bearing prostate tumor cells (LnCap). (Abbreviations: T= tumor, K= kidney, BL= bladder). Animal experiments were performed in accordance with the European Communities Council Directive of November 24^th^, 1986 (86/609/EEC) and the German law for animal welfare and were approved by the local ethical committee for animal experiments.
